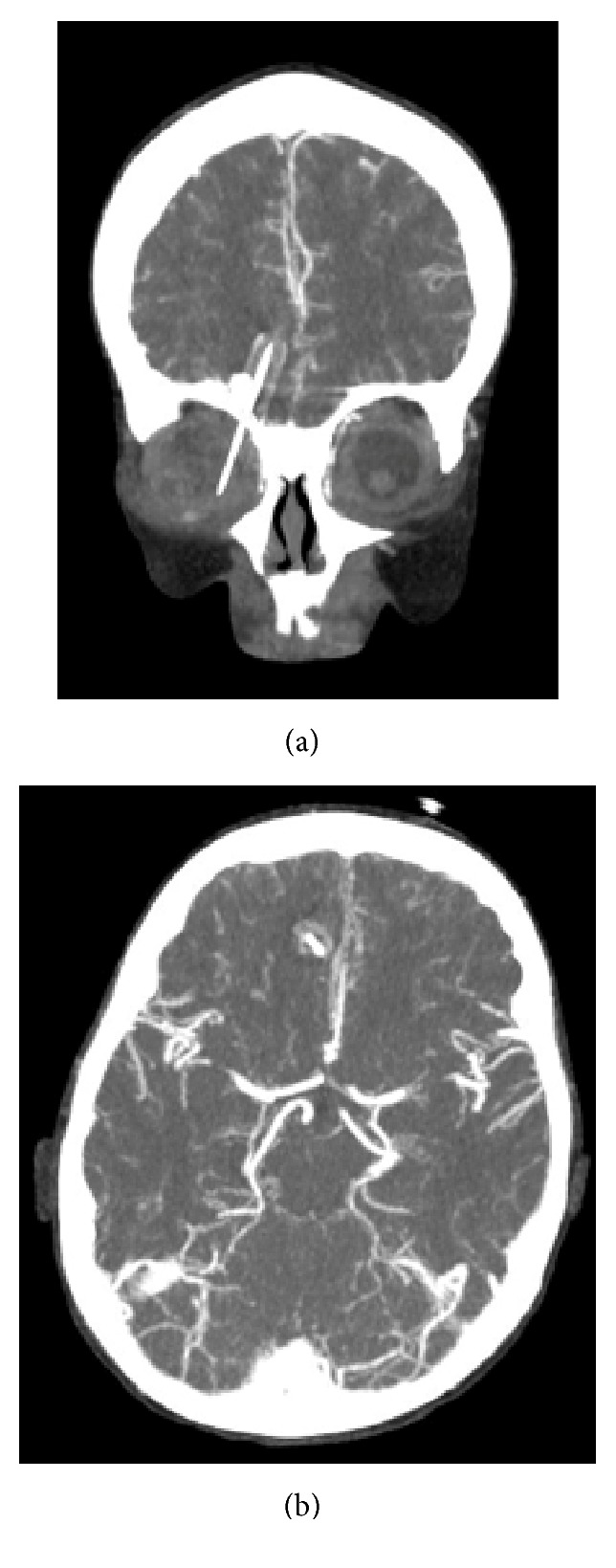# Transorbital Craniocerebral Penetration by a Sharp Object with an Intact Globe

**DOI:** 10.1155/2018/3575897

**Published:** 2018-03-21

**Authors:** Abdullelah A. Alamri, Ahmed R. Algethami, Faisal Alghamdi

**Affiliations:** ^1^Pediatric Emergency Department, King Fahd Medical City, Riyadh, Saudi Arabia; ^2^Ophthalmology Department, King Fahd Medical City, Riyadh, Saudi Arabia

## Abstract

Traumatic eye injuries are common in children and are mostly superficial. Vigilance must always be practiced when examining these children to avoid missing any hidden serious injuries that may result in devastating complications. We describe the acute and definitive management of a child treated 17 h after transorbital craniocerebral penetration by a sharp object. Despite the rarity of these types of injury, a good outcome can be achieved if they are promptly recognized and managed.

## 1. Introduction

Penetrating eye injuries account for about 45% of all pediatric eye traumas. The skulls of children are softer and thinner than those of adults. Moreover, their orbital walls are thinner than other regions of the facial bones, which make them a portal via which slow-penetrating foreign bodies can access the cranial cavity. The risks of late complications increase with organic or wooden foreign objects, such as tree branches or pencils [1].

A significant number of pediatric patients present to the emergency department with trauma. Transorbital penetrating craniocerebral injuries account for 24% of penetrating head injuries in adults and around 45% of such injuries in children [2, 3]. Many types of foreign body have reportedly entered the cranial compartment, and various transorbital routes of entry have been described [4]. Wooden foreign bodies, which usually enter as the result of a low-velocity puncture, are more elusive and have a low threshold for imaging. The radiologic appearance of dry wood on computed tomography (CT) scanning is similar to that of air, and the appearance of hydrated wood is similar to that of soft tissue, rendering detection difficult [5]. In addition, wooden foreign bodies carry significant risks of orbital infection or intracranial abscesses because their porous nature and proximity to soil make them an ideal bacterial reservoir [4, 6–8]. Other complications can also arise, such as meningitis, cerebrospinal fluid leakage, hemorrhage, neurologic deficit, and mortality [1, 5]. Cerebral infection is the most common cause of mortality [9].

## 2. Case Presentation

A 3-year-old boy who was medically healthy fell on a pencil while playing at home. The accident was not witnessed by his family. He was brought to our hospital 17 h after the injury (he visited two other community hospitals that did not have the facilities to deal with such injuries).

**Figure 1:**
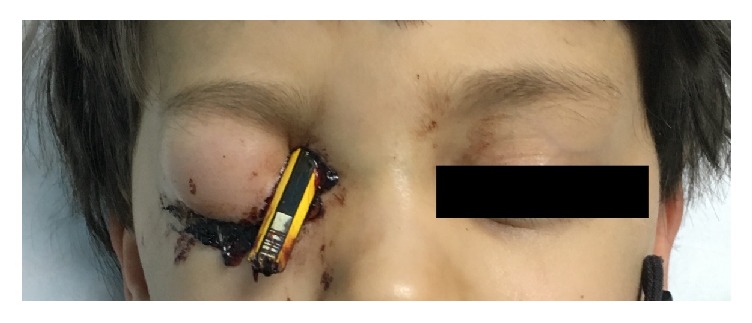


On examination, a sharp object (pencil) was found to be intruding into the right mediosuperior corner of the orbit and oriented diagonally toward the brain, as shown in Figure 1. His Glasgow coma scale score was 15/15, and his vital signs were within normal range. He was not in pain, and he recalled what had happened with no loss of memory. There were no signs of high intracranial pressure. No active bleeding or purulent discharge was visible.

The patient's right eyelid was swollen, and the eye was initially difficult to assess. After primary stabilization, the child was taken for an urgent CT of the brain and orbit with angiography. This revealed that the pencil was nearly 6 cm in length and extended from the right orbit into the ipsilateral frontal lobe (Figures 2(a) and 2(b)). It had entered the orbit caudocranially, piercing the medial aspect of the roof of the orbit and creating a fracture fragment. Within the orbit, it was close to the medial wall of the globe, the walls of which were intact. The intracranial segment of the pencil measured nearly 2.3 cm, and its tip was a few millimeters from the anterior cerebral artery, which was also intact. A small hemorrhage was evident along the brain parenchyma-pencil interface, as was a rim of hypodensity, indicating edema. The circle of Willis was unremarkable, and laboratory test results were normal.

The child was prepared for urgent surgical removal of the foreign body by an oculoplastic and neurosurgical team. The surgery, which was performed under general anesthesia, was uneventful, and the foreign body was removed without the need for a craniotomy. A dressing was applied at the conclusion of surgery. After admission for observation, the child received 1 week of intravenous vancomycin because methicillin-resistant* Staphylococcus aureus* (MRSA) was cultured from a sample taken at removal. Dressings were changed frequently to prevent local infection and cellulitis. His postoperative recovery was uneventful, without neurologic or eye sequelae. A follow-up brain CT was performed after 2 weeks, and it revealed a small contusion with no signs of hemorrhage or infarction. Oral cloxacillin was administered for 1 month, and a 6-month follow-up examination in the neurosurgery and ophthalmology clinic showed an excellent outcome.

## 3. Discussion

The orbit is composed of thin bony walls. These walls are thinner in children than in adults and can easily be fractured by low-velocity penetrating foreign bodies, as seen in this case [10]. Suspended within the orbit, the globe is fairly resilient to trauma because it has a tough sclera and is relatively mobile within a bed of intraorbital fat. Because of this mobility, penetrating orbital injuries can occur without accompanying damage to the globe itself and can easily be overlooked. Foreign bodies can access the cranial cavity via various routes, each of which is associated with damage to certain intracranial structures. Some of these injuries can extend as far as the cerebellum and brain stem [11, 12] depending on the route of access, such as the orbital walls, optic canal, or superior orbital fissure and foreign body trajectory [13, 14]. The most common routes of entry are the superior orbital wall [10, 14] and superior orbital fissure [1, 15, 16]. In general, the outcome of this type of injury depends on the degree and type of damage caused by the foreign body. However, early recognition can prevent life-threatening complications. An initial evaluation with noncontrast CT should be performed to define globe integrity, vascular and bony trauma, and foreign body trajectory and to investigate the occurrence of intracranial penetration and complications. Prompt CT angiography is indicated when the integrity of intracranial circulation requires evaluation and to guide the approach to foreign body removal [17].

Miller II et al. [7] reviewed 42 case reports involving wooden foreign bodies: they reported a mortality rate of 25% after antibiotic treatment and an infection rate of 64%. The cause of death was brain abscess in 57% patients, meningitis or cerebritis in 14% patients, and intracranial hemorrhage in 29% patients. Wooden fragments not only pose a risk to adjacent neurovascular structures in terms of physical injury but also are also associated with a significant risk of infection. Based on several case reports and small case series, the most common site of cerebral abscess formation is around the distal tip of the foreign body [10]. For these reasons, prompt recognition and removal of the fragment are critical [5]. Wooden foreign bodies are prone to microbial contamination because they are a more attractive medium for bacterial and fungal growth than metal [7, 9]. In our patient, MRSA was isolated from a culture of the pencil tip. Retained wooden foreign bodies can result in prolonged suppuration with a draining fistula, panophthalmitis, foreign body granuloma, and brain abscess [6, 8].

In conclusion, penetrating orbital injuries caused by wooden fragments that extend into the intracranial space are rare but potentially life-threatening. Prompt surgical evaluation and treatment with appropriate antibiotic administration are essential and can result in excellent recovery of ocular function and the preservation of neurovascular integrity.

## Figures and Tables

**Figure 2:**